# Identification of 2,4-diacetylphloroglucinol production in the genus *Chromobacterium*

**DOI:** 10.1038/s41598-023-41277-0

**Published:** 2023-08-31

**Authors:** Eric T. Johnson, Michael J. Bowman, Raylane Pereira Gomes, Lilian Carla Carneiro, Christopher A. Dunlap

**Affiliations:** 1 United States Department of Agriculture, Agricultural Research Service, National Center for Agricultural Utilization Research, Crop Bioprotection Research Unit, 1815 North University St, Peoria, IL 61604 USA; 2 United States Department of Agriculture, Agricultural Research Service, National Center for Agricultural Utilization Research, Bioenergy Research Unit, 1815 North University St, Peoria, IL 61604 USA; 3 Institute of Tropical Pathology and Public Health, Federal University of Goiás, Goiânia, Goiás Brazil

**Keywords:** Evolution, Microbiology

## Abstract

The compound 2,4-diacetylphloroglucinol (DAPG) is a broad-spectrum antibiotic that is primarily produced by *Pseudomonas* spp. DAPG plays an important role in the biocontrol disease suppressing activity of *Pseudomonas* spp. In the current study, we report the discovery of the DAPG biosynthetic cluster in strains of *Chromobacterium vaccinii* isolated from Brazilian aquatic environments and the distribution of the biosynthetic cluster in the *Chromobacterium* genus. Phylogenetic analysis of the phlD protein suggests the biosynthetic cluster probably entered the genus of *Chromobacterium* after a horizontal gene transfer event with a member of the *Pseudomonas fluorescens* group. We were able to detect trace amounts of DAPG in wild type cultures and confirm the function of the cluster with heterologous expression in *Escherichia coli*. In addition, we identified and verified the presence of other secondary metabolites in these strains. We also confirmed the ability of *C. vaccinii* strains to produce bioactive pigment violacein and bioactive cyclic depsipeptide FR900359. Both compounds have been reported to have antimicrobial and insecticidal activities. These compounds suggest strains of *C. vaccinii* should be further explored for their potential as biocontrol agents.

## Introduction

The compound 2,4-diacetylphloroglucinol (DAPG) is a broad-spectrum antibiotic that was originally isolated from a fluorescent *Pseudomonas* strain^1^. DAPG is synthesized by a type III polyketide synthase cluster comprising the *phlABCDE* operon^2,3^. The synthesis is initiated by the *phlD* gene, which produces phloroglucinol from malonyl-CoA^2^. Phloroglucinol is subsequentially acylated to produce monoacetylphloroglucinol (MAPG), and a second acylation leads to the production of DAPG^4^.

Subsequently, this molecule can be used to suppress disease causing organisms in soil and is an important component of “disease suppressive soils”^5^ and in biocontrol applications^6^. DAPG is highly effective in controlling the causal agent of “take-all decline” in wheat (*Gaeumannomyces tritici*)^7^ and “tobacco black root rot” (*Thielaviopsis basicola*)^8^. In addition to controlling these fungal ascomycetes, DAPG has been reported to inhibit the oomycete *Pythium ultimum*^9^, the Gram negative bacterium *Erwinia carotovora* subsp. *atroseptica*^10^ and the Gram-positive bacterium *Clavibacter michiganensis* subsp. *michiganensis*^11^. The ecology and use of DAPG in biocontrol applications remains under intensive study^12–14^.

*Chromobacterium* species belong to the Gram-negative class of bacteria, Betaproteobacteria. Strains of *Chromobacterium* have been studied for many reasons such as their ability to be an opportunistic pathogen^15^ and as a model system for quorum sensing in Gram-negative bacteria^16^. *Chromobacterium* have been a rich source of bioactive molecules^17–19^. Recently, they have been used and developed as biocontrol agents to control insect pests^20,21^ and to control plant pathogens^22–25^.

In this study, while exploring the diversity of *Chromobacterium* in a Brazilian river system, we identified strains of *Chromobacterium vaccinii* that possessed a polyketide synthase biosynthetic cluster with homology to the *phlACBDE* operon^26^. This provided motivation to determine the distribution of this cluster in *Chromobacterium* spp. and confirm the bioactivity of the cluster.

## Materials and methods

### Strains

The type strain of *C. vaccinii* DSM 25150^T^ was obtained directly from the DSMZ-German Collection of Microorganisms and Cell Cultures. *Chromobacterium vaccinii* CR1 and CR5 were previously isolated from aquatic environments in Brazil^26^. Both strains were isolated from raw surface water, which was collected directly in sterile flasks from the body of water between 0 and 30 cm water depth, in which the collection and preservation of the samples occurred in accordance to the *National Collection Guide and preservation of samples: water, sediment, aquatic communities and liquid effluents*^27^. *C. vaccinii* CR1 was isolated from João Leite stream, Goiânia, Goiás, Brazil—Geographic location 16° 34′ 30.54" S; 49° 13′ 55.02" W. *C. vaccinii* CR5 was isolated from the Meia Ponte river, Goiânia, Goiás, Brazil—geographic location 16° 54′ 16.3'' S; 49° 07′ 37.8'' W. The isolation details were according to Gomes et al.^28^.

The aquatic environment where strains CR1 and CR5 were isolated from is a river and its tributary. These waters are also impacted by many anthropological pressures, such as industrial, agro-industrial activities (livestock and an intense production of horticultural products), in addition to being used for leisure. The climate of the basin is tropical with two well-defined seasons, dry and rainy, belonging to the Cerrado biome^28^.

### Genome mining for secondary metabolites and DAPG cluster distribution

The website version of the antiSMASH 6.0 software was used to identify putative secondary metabolite clusters in the genomes of *C. vaccinii* CR1, CR5, and DSM 25150^T^^29^. Clusters that were not reliably assigned in the antiSMASH 6.0 software were manually searched using BLAST software to determine a potential function. To determine the distribution of the DAPG cluster, the cluster (identified as GenBank LQR3212800..LQR32_12830) was used as a reference. These loci were manually searched using BLAST software against all available genomes (as of June 1, 2022) in the *Chromobacterium* genus. In addition to the biosynthetic genes of the DAPG cluster (*phlACBDE*), we searched for the regulatory gene associated with the cluster (*phlF, phlG, phlH*) commonly found in *Pseudomonas* strains, accession: CM001558 was used as a reference.

### Phylogenetic analysis

The core genome for the *Chromobacterium* genus was determined and aligned using the BIGSdb program^30^. *Aquitalea magnusonii* DSM 23134^T^ was included as an outgroup. The phylogenetic tree was constructed using MEGA 11 software^31^. The neighbor-joining tree was determined using the Tamura-Nei model (0.40, gamma distributed with invariant sites). Measures of bootstrap support for internal branches were obtained from 1000 pseudoreplicates. For the PhlD phylogeny, the PhlD protein was identified in the genome of *C. vaccinii* CR1 and searched using BLAST software against all available non-redundant proteins in GenBank. All proteins available (9) in the *Chromobacterium* genus were included in the alignment. Due to the large number of *Pseudomonas* proteins available, only a representative sample (11) were included. A *Streptomyces* sp. protein (PhiD) was used as an outgroup. All alignments were performed using MUSCLE implemented under MEGA 11^31^. The alignment was model tested in MEGA 11 and the Maximum Likelihood method with WAG model was selected. Measures of bootstrap support for internal branches were obtained from 1000 pseudoreplicates.

### Construction of *phlACB* expression vector

Genomic DNA was extracted from 1 ml of an overnight culture of *C. vaccini* DSM 25150^T^ as previously described by^32^. The putative *phlACB* cluster (~ 2.8 KB) was amplified from *C. vaccini* DSM 25150^T^ genomic DNA using the primers cluster F (ATG AAG AAG GCA GGC ATA GTG AGC TAT GGC AG) and cluster R (ATC TTC CAG CAC GAA CTT GTA GGC GTA TTG CCA) using Invitrogen Platinum SuperFi II DNA polymerase (Thermo Fisher Scientific, Waltham, MA) according to the manufacturer’s instructions. The gel purified PCR product was reamplified with new primers containing the restriction sites Nco I (5ʹ end) and Hind III (3ʹ end) for cloning into the pET28b expression vector. Following ligation, three independent expression clones with the *phlACB* cluster were identified from plasmids that had been transformed into *E*. *coli* DH 5α. The correct sequence of the amplified *phlACB* cluster in each clone was verified by comparison to the genome sequence of *C. vaccini* CR1 following sequencing of each clone using a MiSeq DNA sequencer. The plasmid clones *phlACB.1, phlACB.3* and *phlACB.5,* as well as empty vector pET28b*,* were individually transformed into BL21 *E*. *coli*.

### Metabolism of DAPG

Each *E. coli* BL21 *phlACB* cell line, as well as the control BL21 cells with pET28b, was grown overnight in LB broth with kanamycin (50 µg/ml). The overnight culture was then used to inoculate M9 minimal medium (200 µl of inoculum into 10 ml media) with kanamycin (50 µg/ml) and grown at 37 °C with shaking (180 RPM) until the OD 600 reached 0.6. Afterwards, each culture received 1 mg of DAPG (dissolved in ethanol, Cayman Chemical, Ann Arbor, Michigan) and Isopropyl-β-d-1-thiogalactopyranoside (IPTG) for a final concentration of 0.2 mM, and incubated at 30 °C Aliquots of the cultures were removed at various time points and stored at 4 °C.

### LC–MS analysis of *Chromobacterium* metabolites

For each time point (1, 2, 3, 4, and 7 days) and each media condition (LB or KMB), 5 μL of *Chromobacterium* culture media was analyzed by LC–MS using a Vanquish HPLC equipped with an ODS-18 column (3 mm × 150 mm, 3 μm particle size, Inertsil, GL Sciences, Inc., Torrance, CA) elution of analytes was accomplished by a linear gradient form 5:95–95:5% 18 MΩ water + 0.1% formic acid: Optima grade methanol + 0.1%formic acid at a flow rate of 0.550 ml/min over 30 min at 50 °C. Column effluent was monitored by UV at wavelengths 254 nm, 280 nm, and 270 nm prior to introduction into an Orbitrap ID-X- tribrid mass spectrometer (ThermoElectron, West Palm Beach, CA) under Xcalibur 4.4 control. Mass spectral data from 200 to 2000 Da m/z were collected in the orbitrap at 120,000 resolution. Tandem mass spectral data of target metabolites was collected using higher-energy collision dissociation (HCD, CE = 25 and 35) in the Orbitrap analyzer.

### Analysis of *E. coli* cultures expressing *phlACB*

Aliquots of *E. coli* media at times 8, 20.5, 28.5, 48, and 68 h from three transformants (pET28-*phlACB*-1, -3, -5) and one plasmid-only control (pET28) were centrifuged at 10,000 RPM for 10 min. A sample of the supernatant (250 μL) was removed and diluted with 250 μL 18 MΩ water. A portion of this dilution (5 μL) was analyzed by LC–MS under the same LC conditions listed above, with the only modification to the mass spectrometric analyses was to lower the mass of detection to 100–1000 Da m/z to allow for the detection of phloroglucinol and MAPG. Authentic standards for DAPG, and phloroglucinol (Acros Organics, Geel, Belgium) were analyzed at 100 μg/ml to establish retention times and MS^2^ fragmentation patterns of expected products. Based on the manufacturer's specifications the level of detection of these analytes is approximately 100 pg/ml under these conditions.

## Results

### Genome mining environmental strains of *Chromobacterium vaccinii*

*Chromobacterium vaccinii* strains CR1 and CR5 were originally isolated from aquatic environments in the state of Goiás, Brazil^26^. The type strain of *C. vaccini* DSM 25150^T^ was also included in the analysis^33^. Table 1 shows five clusters were found across all three strains. The antiSMASH software was only able to assign the function of the violacein and the potential DAPG clusters. A large 68.4 kb trans-AT-PKS cluster was identified using BLAST software to be the biosynthetic cluster of cyclic depsipeptide FR900359^34^. The final two small clusters (6.9 and 8.6 kb) possess domains that suggests they are probable siderophores.

**Table 1:** Biosynthetic clusters identified in *Chromobacterium vaccinii* strains.

Class	Size (kb)	Function	CR1	CR5	MWU205^T^
NRPS	10.8	DAPG	X	X	X
transAT-PKS	68.4	FR900359	X	X	X
Indole	9.2	Violacein	X	X	X
NRPS	8.6	Probable siderophore	X	X	X
T3PKS	6.9	Probable siderophore	X	X	X

### Distribution of biosynthetic clusters in the *Chromobacterium* genus

**Figure 1:**
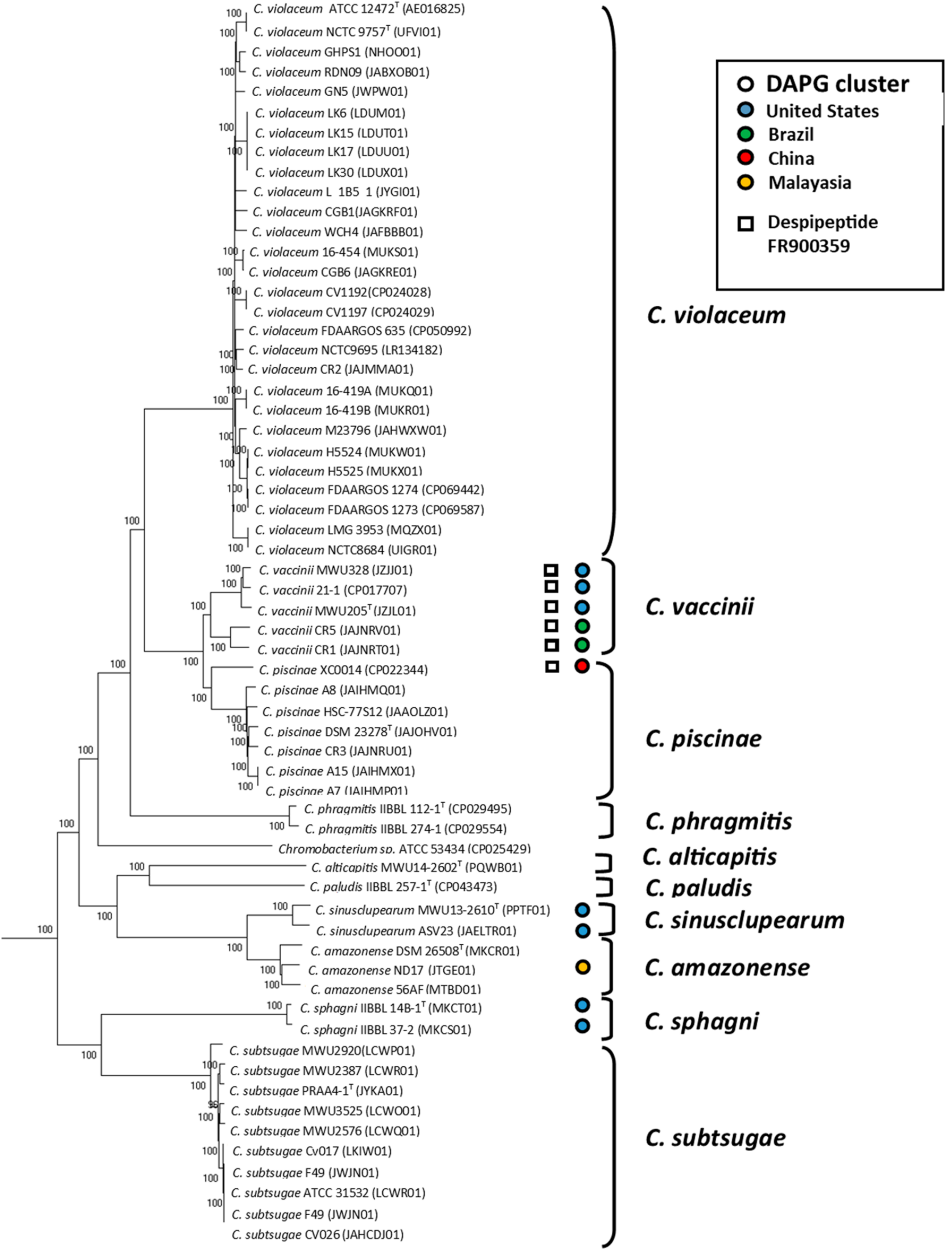
Distribution of DAPG and depsipeptide FR900359 biosynthetic clusters in *Chromobacterium* genus. The phylogeny is based on the core genome of the strains listed in the figure. Measures of bootstrap support for internal branches were obtained from 1000 pseudoreplicates. *Aquitalea magnusonii* DSM 23134^T^ was used as an outgroup and not shown.

The initial discovery of the potential DAPG biosynthetic cluster in genomes of select *C. vaccinii* strains prompted the exploration of the distribution of this cluster in the *Chromobacterium* genus, given the historical importance of this molecule that is normally found in *Pseudomonas* spp. strains^12^. After a broader investigation using all available *Chromobacterium* genomes, the cluster was identified in 11 genomes in the genus that appeared in three main clades (Fig. 1). The results show the cluster was found in all *C. vaccinii* genomes and a closely related *Chromobacterium piscinae* strain. A second clade containing the cluster was identified in *Chromobacterium sphagni.* The third clade was consists of *Chromobacterium amazonense* and a closely related, recently described new species of *Chromobacterium sinusclupearum*^35^*.* The cluster had a broad geographical distribution with seven of the strains from the United States^36–38^, two from Brazil^26^, one from China (CP022344), and one from Malayasia^39^.

All the biosynthetic genes of the DAPG cluster (*phlACBDE*) were found in all of the *Chromobacterium* strains reported to contain the cluster. Interestingly, the *Chromobacterium* strains do not contain all the regulatory genes associated with this cluster in *Pseudomonas* strains. Typically, the DAPG cluster in *Pseudomonas* strains contain the *phlF, phlG* and *phlH* genes, which are known to regulate the expression of the cluster^12^. The *Chromobacterium* strains only contain the *phlF* gene and lack the *phlG* and *phlH* genes, which suggests they utilize a different regulatory mechanism than in *Pseudomonas* strains.

Additionally, the biosynthetic cluster for the depsipeptide FR900359 was also confined to the *C. vaccinii* genomes and one very closely related strain, *C. piscinae* XC0014. The violacein biosynthetic cluster was found in all genomes in Fig. 1, except for the two *Chromobacterium phragmitis* genomes.

### Phylogeny of DAPG cluster

**Figure 2:**
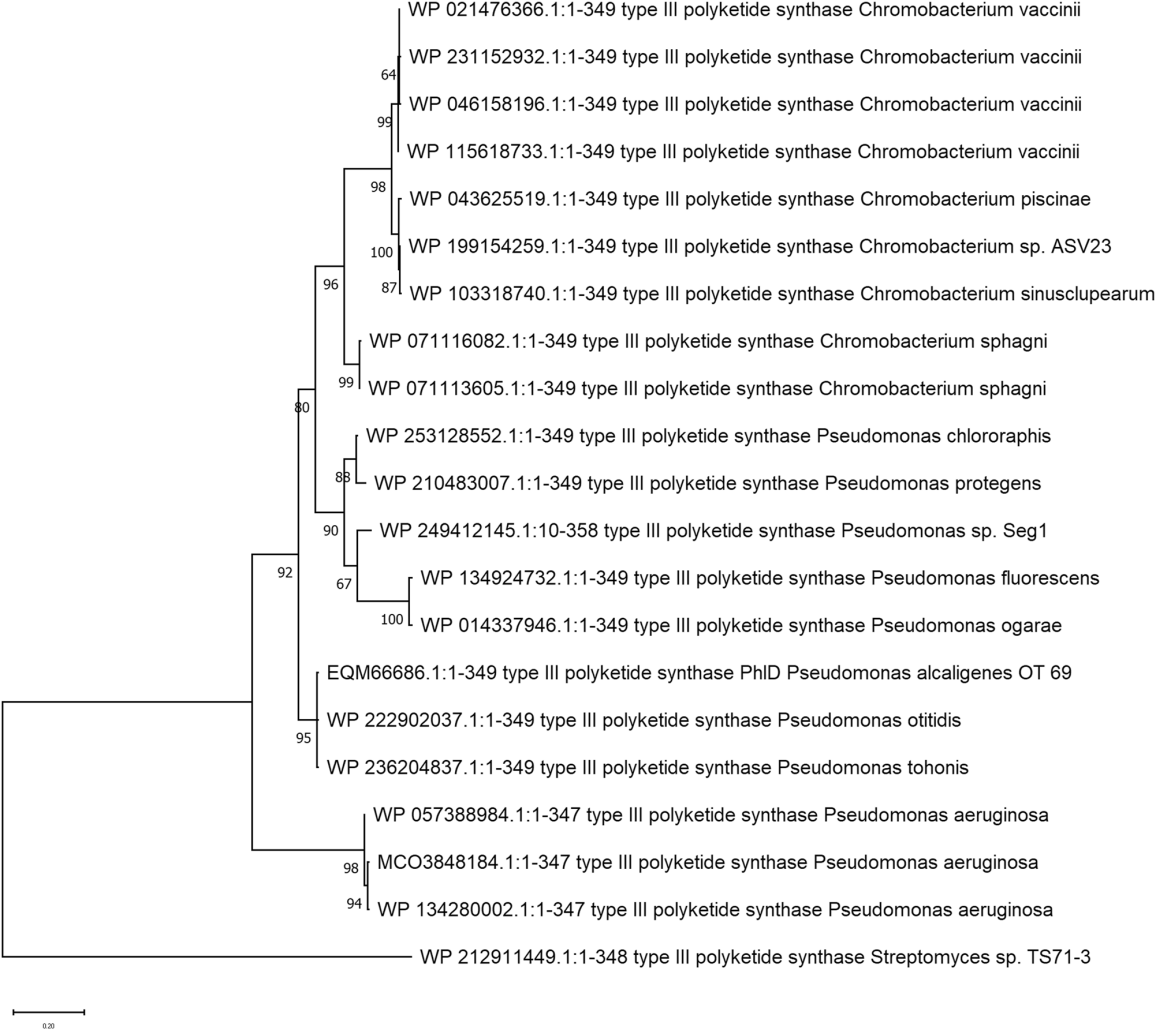
Maximum likelihood phylogeny of PhlD protein from the DAPG biosynthetic cluster. Measures of bootstrap support for internal branches were obtained from 1000 pseudoreplicates.

A phylogeny of the polyketide synthase protein PhlD, which is fundamental to the DAPG cluster, was created to understand the possible origin on the cluster. Figure 2 shows the cluster was closely related to strains in the *Pseudomonas fluorescens* group^40^*.* The average amino acid identity between the proteomes of *C. vaccinii* CR1 and the closest *Pseudomonas* proteome was 47% while the average amino acid identity between two DAPG clusters was 80%. In all *Chromobacterium* genomes containing the cluster, the cluster was in the same physical region of the genome and shared the same flanking regions. A survey of *Chromobacterium* genomes lacking the cluster did not find any alternative genes or clusters at this insertion site.

### Confirmation of biosynthetic cluster assignment and activity

**Figure 3:**
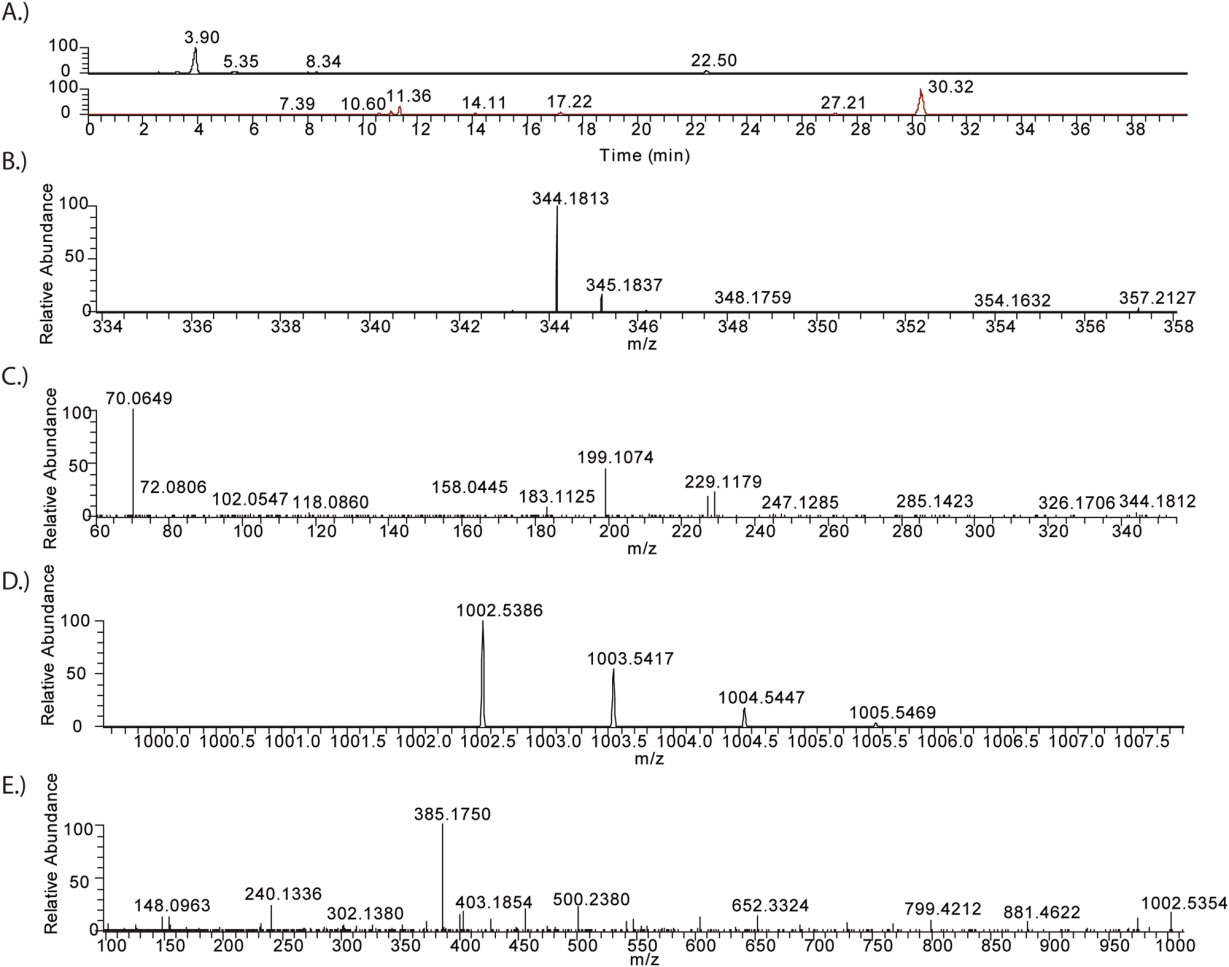
LC–MS data from *Chromobacterium vaccinii* CR1. (**A**) Extracted ion-chromatograms of violacein (black-*m*/*z* 344.1–344.2 Da); and depsipeptide FR900359 (brown-*m*/*z* 1002.5–1002.6 Da); (**B**) mass spectrum of violacein; (**C**) tandem mass spectrum of violacein; (**D**) mass spectrum of FR900359; (**E**) tandem mass spectrum of FR900359.

To confirm the assignment and activity of the biosynthetic clusters LC–MS/MS was used to confirm the presence of the metabolites in culture. The three assigned biosynthetic clusters were violacein, depsipeptide FR900359, and DAPG. Figure 3 shows confirmation for the assignment of violacein and depsipeptide FR900359 using high accuracy mass spectrometry. For all three strains the violacein signal was strong and the depsipeptide FR900359 was much weaker. A very weak signal for DAPG was observed in the strains, which was consistent with the retention time and mass spectrometry of a DAPG reference standard. Based on the results, it was assumed the DAPG cluster was active but not highly expressed under these culture conditions. We evaluated DAPG production under a variety of culture conditions (71 different carbon sources using Biolog plates) to determine if we could improve expression of DAPG activity. Under all the growth conditions tested, none of the conditions significantly improved the production of DAPG.

### Confirmation of DAPG cluster activity through heterologous expression in *E. coli*

**Figure 4:**
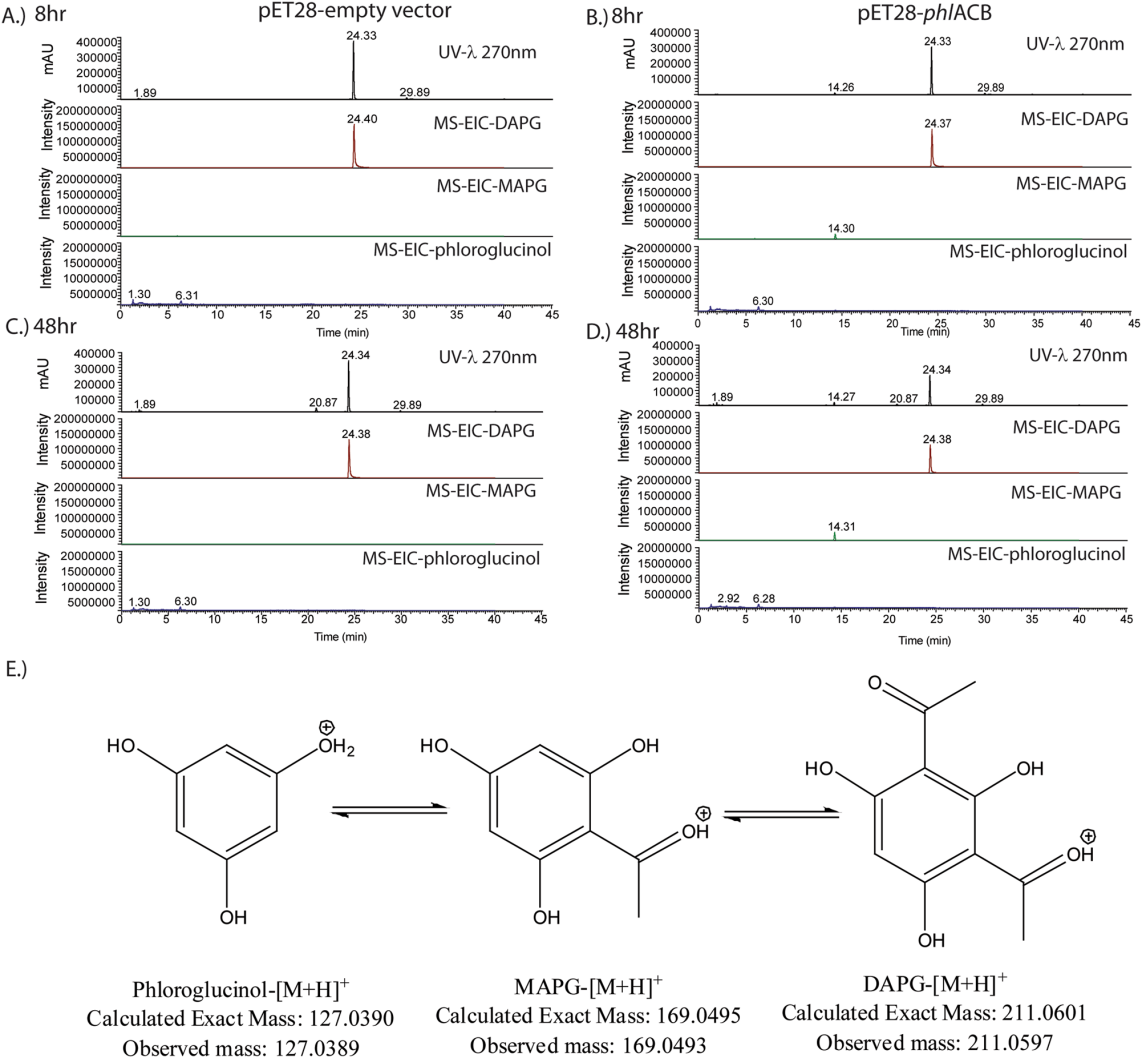
LC–UV–MS data of control and transformed *E. coli* incubated with 100 μg/ml DAPG. Culture media was diluted 1:1 with 18MΩ water prior to analysis. Black-UV at 270 nm (λ_max_ for DAPG); brown-extracted ion chromatogram from 211.05 to 211.07 m/*z*; green-extracted ion chromatogram from 169.00 to 169.20 m/*z*; blue-extracted ion chromatogram from 127.03 to 127.05 m/*z*. DAPG at 24.3 min, MAPG at 14.3 min, phloroglucinol at 2.9 min. (**A**) 8 h control. (**B**) 8 h *phlACB.* (**C**) 48 h control. (**D**) 48 h *phlACB.* E. Structures, masses, and pathway of DAPG degradation.

To confirm the activity of the *Chromobacterium phlACB* gene cluster in the metabolism of DAPG, the cluster was transformed into *E*. *coli*. It should be noted that expression of the *phlACB* genes from *Pseudomonas fluorescens* in *E. coli* resulted in the conversion of phloroglucinol into 2-acetylphloroglucinol (MAPG, 20%) and 3% DAPG in the culture medium^2^. Initial testing of *E*. *coli* expressing *phlACB* from *Chromobacterium* did not produce DAPG from phloroglucinol. Achkar et al. also found that incubation of the *E*. *coli* transformant with DAPG led to complete conversion of DAPG to MAPG (9%) and phloroglucinol (22%) in 48 h^2^. This motivated us to establish the activity of *phlACB* in the direction of DAPG to phloroglucinol. Therefore, cultures were grown in 100 μg/ml DAPG to determine the activity of the transformation of DAPG to MAPG and phloroglucinol. DAPG biotransformation samples, from triplicate transformations, were analyzed by LC–UV–MS/MS to confirm the identity of the products (Fig. 4). Samples over the course of time during incubation were monitored by UV at 270 nm (top panel of Fig. 4A–D), the maximum absorbance for DAPG. The UV signal and extracted ion chromatogram of DAPG remained constant for the control reaction where plasmid-only *E*. *coli* was the biocatalyst over 48 h (Fig. 4A,C). *E*. *coli* expressing *phlACB* showed a decrease in UV and EIC signal for DAPG over the course of 48 h, while having an increased signal for MAPG (14.30 min as determined by UV and accurate mass, < 1 ppm error, due to the lack of authentic standard) and phloroglucinol (at 48 h, 2.92 min based on accurate mass and comparison with authentic standard). At longer reaction times with *E*. *coli* expressing *phlACB*, DAPG and MAPG signals decreased and additional UV peaks appeared.

## Discussion

DAPG has been an important molecule in the bioactivities of rhizosphere colonizing *Pseudomonas* species and the subject of extensive investigations for 40 years^12^. It is for this reason we found it interesting to discover strains of *C. vaccinii* isolated from aquatic environments have the DAPG cluster^26^. The type strain of *C. vaccinii* and other representative isolates of *C. vaccinii* were originally isolated from the rhizosphere and water environments associated with native and cultivated cranberries (*Vaccinium macrocarpon* Ait.)^33^. This coincidence suggests that DAPG could provide a similar ecological function in *Chromobacterium* as seen in *Pseudomonas* species. In *Pseudomonas* species, DAPG has long been known to suppress phytopathogens and other microbes in the rhizosphere^41^. In addition, it was shown that DAPG containing *Pseudomonas* strains were a key component of disease suppressive soils in controlling the wheat root disease “take-all decline”^5^. It is possible that DAPG in *Chromobacterium* may play a similar ecological role for plants in bogs or similar aquatic environments. It is also noteworthy that these genera are only distantly related, and the closest shared clade is at the phylum level (Proteobacteria) with *Chromobacterium* belonging to the class Betaproteobacteria and *Pseudomonas* belonging to the class Gammaproteobacteria.

The distribution of the DAPG biosynthetic cluster in the *Chromobacterium* genus is confined to a few different clades. It is interesting to find all members of *C. vaccinii, C. sphagni* and *C. sinusclupearum* have this cluster (Fig. 1). However, these species have limited genome representation with 5, 2, and 2 whole genomes sequenced, respectively. This suggests there is a selection pressure to maintain the cluster in the species, notably in *C. vaccinii* where all strains are from diverse environments. The analysis also shows the DAPG cluster has broad geographical distribution with genomes from North America, South America, and Asia. In addition to the DAPG cluster, we identified two other biosynthetic clusters in the genomes of interest, depsipeptide FR900359 and violacein. Most strikingly, depsipeptide FR900359 occurred in tandem with DAPG in the *C. vaccinii* strains while violacein appeared in all *Chromobacterium* genomes with the exception of the two *C. phragmitis* genomes. It is not clear if DAPG and depsipeptide FR900359 are an adaptation to a specific ecological need or if their correlation is a coincidence.

The depsipeptide FR900359 was found to be a powerful and selective inhibitor of G protein-coupled receptors (GPCR) in humans and other mammals^42,43^. GPCRs are important elements for intracellular signal transduction which respond to external ligand binding and initial response cascades through guanosine triphosphate and guanosine diphosphate exchange^44^. Now depsipeptide FR900359 and closely related compounds, collectively known as chromodepsins, are important pharmacological tools for use as therapeutics and tools to study GPCR function^45^. Interestingly, chromodespins were initially isolated from a plant (*Ardisia crenata*) used in traditional medicine^46^. Later, it was discovered the compound was actually produced by an endosymbiotic bacteria belonging to the genus *Burkholderia*^47^. The ecological role of this compound is not well understood. Chromodespins demonstrate a lack of inhibition toward plant GPCRs^43^. It has been suggested that they may provide a benefit to their plant host by acting as a herbivore deterrent^47^. Interestingly, chromodespins have been shown to have insecticidal activity against bean bug (*Riptortus pedestris*), as well as inhibit the GPCRs of the pest insects white fly (*Bemisia tabaci*) and silk moth (*Bombyx mori*)^45^. This suggests strains of *C. vacinnii* may also be useful in the control of insect pests.

All *Chromobacterium* genomes except the two *C. phragmitis* genomes contained the purple pigmented compound violacein. Violacein has been reported to have a variety of bioactive properties. It has been shown to have antifungal properties against a large variety of phytopathogenic and human fungal pathogens^48^. It has been reported to have antibacterial activities^49^ and antinematicidal activities^50^. The compound showed antifeedant, larvicidal and pupicidal activities against Asian armyworm (*Spodoptera litura* Fab.)^51^. These broad-based bioactivities suggest that violacein may also contribute to the biocontrol potential of these *Chromobacterium* strains.

The phylogeny of the PhlD protein (Fig. 2) suggests all DAPG clusters in *Chromobacterium* share a common ancestor and the closest non-*Chromobacterium* ancestor likely originated in the *Pseudomonas flourescens* group. From the phylogenetic tree it is difficult to definitively infer if the cluster had one transfer event into *Chromobacterium* and some descendent lineages lost the biosynthetic cluster or if other horizontal gene transfer (HGT) events occurred within the *Chromobacterium* genus (e.g. a HGT from *C. vaccinii* to *C. sinusclupearum*).

In wild type *C. vaccinii* isolates, the production of DAPG could only be detected in trace amounts. Efforts to increase the production of DAPG through screening a variety of media conditions and carbon sources were unsuccessful. It may be possible that molecules in aquatic environments induce the biosynthesis of DAPG in *Chromobacterium*. Nevertheless, we confirmed that the biosynthetic genes in *Chromobacterium* for DAPG production were intact and functional. Our results also suggest the regulatory mechanism of *Chromobacterium* strains is significantly different than *Pseudomonas* strains since they lack the known regulatory genes *phlG* and *phlH*^12^. In addition, they lack the *Pseudomonas* post translational regulatory genes *rsmA* and *rsmE* (data not shown)^12^. More experiments will need to be performed in the future to discover the regulatory mechanism of DAPG production in *Chromobacterium*. We assumed the lack of activity was due to the lack of expression of these genes. We transferred the *phlACB* cluster into *E. coli* to have direct control over the expression of these genes. Initial testing of the transformant failed to produce DAPG from phloroglucinol. This is consistent with previous observations that expression of the *phlACB* cluster in *E. coli* produced no or trace amounts of DAPG^2,52^. However, in past studies the reverse reaction, conversion of DAPG to MAPG and phloroglucinol was active^2,52^. Monitoring of the reverse reaction showed a time-dependent decrease in the amount of DAPG present in the media when *phlACB* was expressed. There was an increase in the UV_270nm_ at retention times corresponding to the exact mass of MAPG and phloroglucinol (Fig. 4E) demonstrating the cluster could convert DAPG to MAPG and phloroglucinol. It should be noted in experiments where *E*. *coli* expressing *phlACB* transformants were incubated with phloroglucinol in an attempt to produce DAPG, the phloroglucinol used contained a small DAPG (< 1%) impurity. When incubated with the *E*. *coli* expressing *phlACB* transformants, this DAPG impurity was converted to phloroglucinol and no reaction was observed in the direction of DAPG formation. It is unknown why *phlACB* transformants favored the production of phloroglucinol and the biotransformation to DAPG did not occur. However, Liu et al.^52^ recently demonstrated enhanced production of DAPG in *E*. *coli* expressing *phlACB* transformants through co-expression of *acc, marA* and *phlE* genes.

A survey of literature identified a few examples of strains of *Chromobacterium* being evaluated as potential biocontrol agents to control plant pathogens^22–25^. Two of the studies use *Chromobacterium haemolyticum* C-61, which displayed strong antifungal activities against several plant pathogens^23,24^. These studies identified another novel cyclic lipopeptide which they named chromobactomycin, which was responsible for the observed activity^24^. The genome is available for this strain, and it does not contain the DAPG cluster. Han et al. identified *Chromobacterium* sp. JH7 during a screening assay to identify antagonists of *Cylindrocarpon destructans*, a root rot pathogen of ginseng^22–25^. The authors were not able to identify the strain to the species level. It is possible that DAPG or depsipeptide FR900359 may be associated with this activity, but there is currently no data to support this speculation. In a recent study, *C. vaccinii* was shown to inhibit several fungal plant pathogens through emission of bioactive volatiles^22–25^. We could not find any reported DAPG activity in *Chromobacterium* sp. in the literature.

Our results confirm the presence and activity of the DAPG biosynthetic cluster in the *Chromobacterium* genus. These findings and the limited investigations of the biocontrol activity against plant pathogens by the *Chromobacterium* genus suggests additional research in this area is warranted.

## Data Availability

All data generated or analyzed during this study are included in this published article. All sequence data generated by our laboratory are available at https://www.ncbi.nlm.nih.gov/bioproject/PRJNA782632.
